# Impact of day-surgery on cancer treatment costs: a city-level case study in Ningxia, China

**DOI:** 10.1186/s12913-026-14323-6

**Published:** 2026-03-30

**Authors:** Yiwen Guo, Rui Xing, Elizabeth Maitland, Stephen Nicholas, Rugang Liu, Chuanchuan Xu

**Affiliations:** 1 Laboratory for Digital Intelligence & Health Governance, Nanjing Medical University, Nanjing, 211166 China; 2 School of Health Policy & Management, Nanjing Medical University, Nanjing, 211166 China; 3 Jiangsu Provincial Institute of Health, Nanjing Medical University, Nanjing, 211166 China; 4 School of Management, University of Liverpool, Chatham Building, Chatham Street, Liverpool, L697ZH England; 5 Health Services Research and Workforce Innovation Centre, Newcastle Business School, University of Newcastle, Newcastle, NSW Australia; 6 Australian National Institute of Management and Commerce, Sydney, Australia; 7 School of Humanities and Management, Ningxia Medical University, Yinchuan, China

**Keywords:** Day-surgery management model, Tumor treatment costs, Time-based regression discontinuity design, Healthcare resource optimization, Medical cost control

## Abstract

**Background:**

Aiming to reduce hospitalization expenses through shorter stays and improved resource allocation, China’s national day surgery cancer treatment reforms were implemented by local health authorities. This study evaluates the impact of the day-surgery management model implemented in Ningxia in 2019 on cancer hospitalization costs, including treatment, comprehensive, diagnostic, consumption, and medicine costs.

**Methods:**

From January 2015 to December 2022, this retrospective study analyzed data on cancer hospitalization costs from municipal hospitals in Yinchuan, Ningxia. Data on 122,252 patients were collected, excluding deceased patients, incomplete records, and waived costs. Patient data included sex, age, occupation, payment method, admission date, clinical condition, and hospitalization costs. Statistical analysis used STATA, with χ² tests and Fisher’s exact tests comparing patient characteristics pre- and post-implementation of the day-surgery model. A sharp regression discontinuity design (RDD) assessed the model’s impact, dividing observations into pre- (2015–2018) and post-implementation (2019–2022) phases. A sensitivity analysis controlling for length of stay (LOS) was conducted to verify our cancer hospitalization costs changes.

**Results:**

RDD analysis showed that the day-surgery model had no significant impact on total hospitalization costs (*p* = 0.084). Nevertheless, it was associated with significant reductions in several cost components, significant reductions occurred in comprehensive fees (-RMB293.85), diagnostic fees (-RMB508.3), consumption fees (-RMB386.38), and medicine fees (-RMB1569.5), while treatment fees rose by 46.2%. Crucially, the sensitivity analysis revealed that when controlling for LOS, the total hospitalization cost shifted to a significant increase (+ RMB564.7), and the surge in treatment fees intensified (+ RMB1614.2). This confirms that apparent aggregate cost neutrality masks underlying shifts in cost structure, with savings largely mediated by shorter stays that offset higher daily treatment intensity under the day-surgery model. Diagnostic and comprehensive fee reductions showed time-specific responsiveness, while medicine costs fluctuated at non-policy nodes.

**Conclusion:**

The day-surgery model in Ningxia did not significantly reduce aggregate hospitalization costs, with the lower diagnostic, medicine, consumption and comprehensive fee categories offset by increased treatment costs. These results underscore the complexity of day-surgery economics: efficiency gains from faster turnover were partially offset by technology-driven cost escalations. Simply “improving efficiency” is insufficient, with policymakers and hospitals encouraged to enhance the financial sustainability of day-surgery reforms by targeted treatment cost-containment strategies.

## Key points

1. This study employed a sharp regression discontinuity design (RDD) to assess the impact of the day-surgery management model on total cancer treatment expenses.
2. The findings indicate that while the model did not significantly affect overall hospitalization costs, it altered the cost structure by reducing comprehensive, diagnostic, consumption, and medicine fees, but significantly increasing treatment fees.
3. This highlights the necessity for hospitals to balance operational efficiency with overall cost control, as the rise in treatment costs imposes an additional financial burden on patients.

## Introduction

Initiated at the national level, China’s healthcare reforms, policies and strategies are interpreted, piloted and implemented at different times and under different conditions by local provincial and city health authorities. Launched in 2009, China’s on-going healthcare reform program aims to control medical expenses, optimize the allocation of healthcare resources, ensure high-quality treatment outcomes and reduce the healthcare cost burden on families [1, 2]. These reforms involved the financing of hospitals, setting medical fees for patients and doctors, implementing a universal health security system, including new health insurance schemes, integrating health worker training and addressing rural-urban disparities in healthcare access and resourcing [3]. Different cities and provinces have introduced these national reforms at their own pace and modified the reforms to meet their own resources and capabilities [4]. To assess China’s day-surgery management reform, we evaluate the impact of day-surgery on patient hospital expenses in Ningxia, an autonomous region in northwest China.

Internationally, the day-surgery management model has emerged as an innovative cost-saving approach, optimizing patient admission and discharge times, with shorter hospital stays, typically no more than 24 h (and at most 48 h), reduced hospital costs, and more efficient allocation of medical resources [5–7]. Current research indicates that compared with similar conventional inpatient treatment pathways, day surgery is able to reduce hospitalization costs mainly due to shorter hospital stays and lower management expenses. For hospitals, the day-surgery model increases operating room utilization, enhances surgical throughput, and boosts overall service efficiency [8]. The day-surgery model can not only enhance hospital operational efficiency but also significantly lower patient hospitalization costs, hospital duration and disruption to daily life, leading to increased patient satisfaction [6, 8]. Supported by minimally invasive surgical techniques, patients also benefit from smaller incisions and faster recovery, shortening the rehabilitation period and reducing the risk of hospital-acquired infections [6, 7].

In China, day surgery has transitioned rapidly from independent clinical initiatives to a government-supported strategy [7]. Nationally, the National Health Commission guides hospitals to implement day-surgery, specifying requirements and conditions for its adoption [7, 8]. These policies are also supported by China’s national medical insurance schemes, reimbursing simple to complex surgeries across various specialties [7]. While day-surgery is national policy, day-surgery has been implemented at different times by different provinces, with different payment standards and financial support [7]. Existing literature often lacks the critical financial baseline of detailed, component-wise cost reporting from the day-surgery era, moving beyond qualitative descriptions to a precise numerical assessment of savings or cost shifts. In contrast to research in other countries [9, 10], little attention has been given to day-surgery in China. We address this research gap by analyzing day-surgery’s impact on cancer hospitalization costs in Ningxia’s municipal hospitals after 2019 [11]. Ningxia serves as a highly relevant and representative setting for this analysis. As a mid-sized autonomous region in northwest China, its healthcare system grapples with the universal challenges of cost containment and efficiency improvement, while its provincial-level implementation of national reforms offers a replicable case study for similar regions across China. Municipal level-three hospitals are China’s best resourced and equipped hospitals, with highly trained medical professionals, undertaking both medical training and scientific research and providing inter-provincial medical and health services [12]. For Ningxia’s municipal hospitals, we estimate the day-surgery model’s effect on cancer patient hospitalization expenses, and on the treatment, comprehensive, diagnostic, consumption, and medicin*e* fees components of aggregate hospitalization costs.

## Methods

### Subjects

Using the information management system of municipal level-three hospitals located in the Ningxia’s capital, Yinchuan, we collected data on each hospital’s day-surgery management model for cancer diseases between January 2015 and December 2022. All participants signed the written informed consent form, with the legal guardians of the minor participants signing the consent form and the minor themselves also providing written assent. No participant was adversely affected by refusal or withdrawal from the study. All data from the hospital information system was anonymized, with no individual’s name revealed. Exclusion criteria included cases where patients had deceased, cases with incomplete or inaccurate records, such as missing information on initial diagnosis, primary surgical procedures, or hospitalization costs, and cases where costs were waived.

### Research design

A retrospective methodology was employed to collect data from the hospitals’ information management system on 122,252 patients, including disease classifications and surgical procedure names, with the final diagnostic codes shown in Table 1. Patient medical data collected included sex and age, payment method, admission date, occupation, and hospitalization costs. The patient data classifications are detailed and explained in Table 2.

**Table 1:** Cancer disease diagnoses codes

NO.	Main Diagnosis Code	Main Diagnosis Name
1	Z51.002	Postoperative Radiotherapy for Malignant Tumors
2	Z51.003	Radiotherapy for Malignant Tumors
3	Z51.100	Chemotherapy Course for Tumors
4	Z51.100 × 004	End-stage Chemotherapy for Malignant Tumors
5	Z51.101	Preoperative Chemotherapy for Malignant Tumors
6	Z51.102	Postoperative Chemotherapy for Malignant Tumors
7	Z51.103	Maintenance Chemotherapy for Malignant Tumors
8	Z51.104	Palliative Chemotherapy
9	Z51.200 × 008	Chemotherapy
10	Z51.500 × 003	Maintenance Treatment for End-stage Malignant Tumors
11	Z51.800 × 095	Immunotherapy for Malignant Tumors
12	Z51.800 × 925	Laser Treatment for Malignant Tumors
13	Z51.801	Targeted Therapy for Malignant Tumors
14	Z51.807	Postoperative Targeted Therapy for Malignant Tumors
15	Z51.810	Tumor Immunotherapy

**Table 2:** Variables about patient medical data

Variables	Definition	Classification
Sex	The patient’s biological sex.	1 = Male, 2 = Female.
Age	The patient’s age at admission.	1 = < 30, 2 = (30, 44), 3 = (45, 59), 4 = (60, 74), 5 = ≥ 75.
Payment method	The payment method for medical insurance used by the patient during hospitalization.	1 = Full government expense, 2 = Full self-payment, 3 = Commercial medical insurance, 4 = Basic medical insurance for urban residents, 5 = Basic medical insurance for urban workers, 6 = New rural cooperative medical care (NRCMS), 7 = Poverty relief, 8 = Other social insurance, 9 = Others.
Occupation	The patient’s occupation before admission.	1 = Technical staff, 2 = Enterprise management personnel, 3 = Self-employed, 4 = Government officials, 5 = Students, 6 = Labor workers, 7 = Farmers, 8 = Unemployed, 9 = Others.
Admission data	The specific date of hospital admission.	1 = Before Jan 2019, 2 = After(including) Jan 2019.

Cases were rigorously screened according to the predefined inclusion and exclusion criteria to ensure the integrity of the dataset. We broke down the aggregate hospitalization costs into different types of fees, comprising comprehensive fees, diagnostic fees, treatment fees, consumption fees, and medicine fees. These categories were selected as they constitute the core, mandatory financial reporting framework for hospitalization costs in Chinese public hospitals, as stipulated by the National Health Commission and the Ministry of Finance. They reflect the standard itemized billing structure presented to patients and insurers, ensuring our analysis aligns directly with real-world hospital accounting and reimbursement practices. The definitions and calculation methods for these fees are specified in Table 3. To ensure clarity, we strictly distinguished between fee categories to avoid overlap: ‘comprehensive fees’ cover basic, routine medical services (including nursing, accommodation, and general injections); ‘treatment fees’ encompass specific clinical interventions and technical procedures (such as surgery, anesthesia, and radiotherapy). These categories adhere to the standardized financial classification system of Chinese public hospitals, ensuring they are mutually exclusive.

**Table 3:** Definition of different costs

Cost	Definition	Formula
Comprehensive fees	Costs incurred for basic and routine medical services, including accommodation, nursing care, and general operational procedures (e.g., injections, dressing changes), excluding specific clinical interventions.	Comprehensive fees = General medical service fee + General treatment operation fee + Nursing fee + Other fees
Diagnostic fees	Covers the professional service fees for the entire process of disease diagnosis.	Diagnostic fees = Pathology fee + Laboratory diagnosis fee + Imaging diagnosis fee + Clinical diagnosis fee
Treatment fees	Fees for specific clinical interventions and technical procedures, such as surgery, anesthesia, radiotherapy, and specialized non-surgical therapies.	Treatment fees = Non-surgical treatment fee + Clinical physical therapy fee + Surgical treatment fee + Anesthesia fee + Operation fee + Rehabilitation fee + Traditional Chinese medicine treatment fee
Consumption fees	The costs of various medical consumables used during the medical process.	Consumption fees = Blood transfusion fee + Albumin product fee + Globulin product fee + Coagulation factor product fee + Cytokine product fee + Examination disposable material fee + Treatment disposable material fee + Surgery disposable material fee + Other fees
Medicine fees	The costs incurred for drug treatments.	Medicine fees = Western medicine fee (including antibacterial drug fee) + Proprietary Chinese medicine fee + Chinese herbal medicine fee
Hospitalization costs	All the costs during the treatment process.	Hospitalization costs = Comprehensive fees + Diagnostic fees + Treatment fees + Consumption fees + Medicine fees

### Statistical methods

Excel software was used for data collection, validation, and cleaning, and STATA 17.0 software for statistical analysis. Using frequency counts and composition ratios, descriptive analysis was first conducted on the basic characteristics of the survey subjects. χ² tests and Fisher’s exact probability method were then used to compare the basic characteristics of patients before and after the implementation of day surgery management, with a significance level set at α = 0.05.

Classified into sharp and fuzzy designs, regression discontinuity design (RDD) is a robust method for policy evaluation [13]. Employing a sharp RDD approach, monthly observations were divided into the pre-implementation (January 2015 to December 2018) and post-implementation (January 2019 to December 2022) day-surgery phases. RDD is a quasi-experimental method used to evaluate the causal effects of policies or interventions, which involves setting a threshold on a continuous variable, such as time or age and comparing outcome differences between individuals just above and below this threshold policy implementation date [14]. In the health research area, it is often used to evaluate the effect of a specific health policy [15–17].

In RDD, the running variable is a continuous variable that determines eligibility for an intervention based on a predefined threshold. At the threshold, the likelihood of receiving the intervention “jumps” from a lower probability to a higher one, creating a natural experiment to isolate the intervention’s impact. In our study, the outcome variable Y represents medical treatment costs; the running variable R is the years/months timeline; the cutoff, c, was January 2019, when the day surgery was implemented; and the treatment assignment T_i_, indicates whether a patient received day surgery. At the cutoff c, the probability of receiving day surgery jumps sharply from near 0% to near 100%. This abrupt change allows estimation of the policy’s causal effect on costs specifically for patients near the cutoff, termed the local average treatment effect (LATE) [18–20].

We used a nonparametric covariate-adjusted regression discontinuity design (RDD) with local linear regression (first-degree polynomial) to estimate the policy’s impact on medical costs near the cutoff. To explicitly control for potential confounders and improve estimation precision, we included a vector of covariates Z_i_ (sex, age, occupation, payment method, and diagnostic codes) in the model. The estimation equation was specified as:

$$Yi = \alpha + \tau Di + \beta \left( {Ri - c} \right) + \gamma Di\left( {Ri - c} \right) + \delta Zi + i$$

where Di is the treatment indicator, and τ represents the local average treatment effect. This approach focuses on data closest to the threshold, reducing bias from distant observations [21]. Higher-degree polynomials were avoided due to risks of overfitting and unstable estimates [21, 22]. Bandwidth selection methods, including mean squared error, MSE-optimized, MSERD (bandwidth that minimizes the MSE of the point estimator, where the bandwidth is the same on both sides of the cutoff); MSETWO (bandwidth that minimizes the MSE of the point estimator, where the bandwidths are different on each side of the cutoff ); and MSESUM (bandwidth that minimizes the MSE of the sum of the regression coefficients) were applied.

**Fig. 1:**
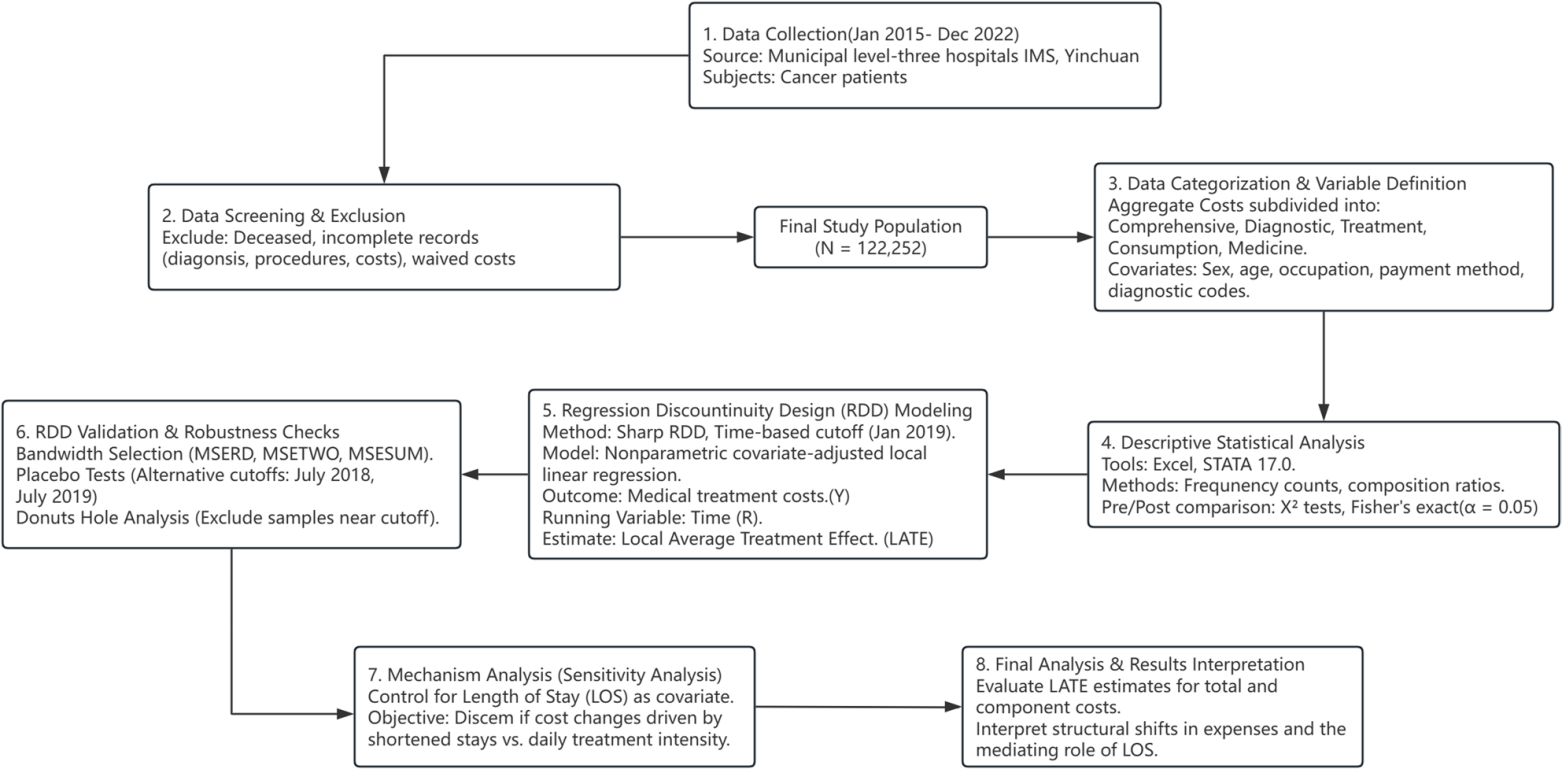
A comprehensive flowchart. The diagram illustrates the sample selection (N = 122,252) and statistical framework, utilizing a Sharp Regression Discontinuity Design (RDD) to evaluate medical cost changes

Finally, to verify the mechanism of cost changes and address the potential mediating role of hospitalization duration, we conducted a specific sensitivity analysis regarding length of stay (LOS). We compared the coefficients of the baseline model against a model where LOS was included as a covariate. This comparison allowed us to discern whether cost reductions were driven by shortened stays or by changes in daily treatment intensity. Additional robustness checks, such as Donut Hole analysis, were also performed to confirm the stability of our results [21, 22] (Fig. 1).

## Results

### Descriptive analysis

Our sample comprised 122,525 day-surgery cancer patients covering 15 categories, including postoperative radiotherapy for malignant tumors and radiotherapy for malignant tumors. As shown in Table 4, the age distribution of patients was primarily concentrated between 45 and 59 years.

**Table 4:** Age distribution and hospitalization expenses of cancer patients

Age	Before 2019				After 2019			
	Number of patients	Proportion (%)	Average length of stay (days)	Average hospitalization cost (RMB)	Number of patients	Proportion (%)	Average length of stay (days)	Average hospitalization cost (RMB)
<30	4376	7.99	9.24	7978.96	2390	3.52	1.23	2566.31
30–44	9954	18.18	9.56	11345.35	8335	12.30	1.25	5225.16
45–59	24,603	44.95	8.89	11169.22	32,095	47.35	1.24	4991.13
60–74	15,008	27.42	9.21	11773.97	23,101	34.08	1.22	4869.38
≥ 75	798	1.46	10.84	14564.07	1866	2.75	1.20	5206.91

Table 5 shows that hospitalization costs, which included all the expenses during the treatment process, were significantly reduced across all age groups after the introduction of day-surgery. Average hospitalization costs in Table 5 varied by payment method, but all fell following the implementation of the day-surgery management model (with the commercial medical insurance sample too small to draw conclusions).

**Table 5:** Different payment methods and hospitalization expenses of cancer patients

Payment method	Non-day			Day-case		
	Number of patients	proportion	Average hospitalization cost (RMB)	Number of patients	proportion	Average hospitalization cost (RMB)
Full government expense	73	0.13	93133.01	18	0.03	3941.03
Full self-payment	8309	15.18	8616.59	20,252	29.88	4462.24
Other	1357	2.48	9153.28	562	0.83	3986.75
Other social insurance	604	1.10	8111.83	123	0.18	3641.00
Commercial medical insurance	4	0.01	3414.87	6	0.01	14910.89
Basic medical insurance for urban residents	23,360	42.68	11267.16	9956	14.69	4624.75
Basic medical insurance for urban workers	7973	14.57	13799.94	31,798	46.91	5345.22
New rural cooperative medical care	13,032	23.81	11348.97	5032	7.42	4501.34
Poverty relief	27	0.05	8623.66	40	0.06	4978.61

Note: Full government expense referred to medical expenses being fully borne by the government; full self-payment meaned all medical expenses are paid by oneself; other payment method included medical assistance (non-poverty alleviation), charitable funds or public welfare assistance and enterprise supplemental medical insurance

### Regression discontinuity analysis results on the impact of day surgery management model on hospitalization costs

The regression discontinuity analysis, validated by covariate continuity checks, indicates that while the day-surgery model did not significantly reduce total hospitalization costs overall, it prompted a marked structural shift within cost components, significantly decreasing comprehensive, diagnostic, consumption, and medicine fees but increasing treatment fees.

#### Applicability assessment

A time-based RDD with January 2019 as the cutoff point was used to evaluate the impact of the day-surgery management model on hospitalization costs. We addressed the issue of endogenous grouping by testing the continuity of covariates before and after the cutoff point [21]. Covariates analyzed include sex, age, medical payment method, occupation, and diagnosis code. Local linear regression was performed to test for continuity at the January 2019 cutoff.

From Table 6, the three covariates—sex, age, occupation, and diagnosis code—exhibit continuity across the cutoff point, as indicated by p-values greater than 0.05. However, the medical payment method displayed discontinuity at the cutoff. Upon further analysis using a fuzzy RDD approach, the medical payment method was found to be continuous at the cutoff point (*p* = 0.417), suggesting no significant change in payment methods. The initial discontinuity is attributed to the treatment of the medical payment method as a continuous variable. We determined that patient grouping was solely determined by the timing of policy implementation, and confirmed that there are no abrupt changes in patient characteristics, such as age and disease severity, before and after the policy. RDD was suitable for assessing the true impact of the day surgery model on hospitalization expenses for cancer patients.

**Table 6:** Continuity test of covariates

Concomitant variable	bandwidth	Parameter	Coefficient	Standard error	Z	*P*	95%CI
Age	MSERD	Conventional	0.382	0.334	1.145	0.252	[-0.272, 1.037]
	MSERD	Robust	-	-	0.814	0.415	[-0.455, 1.103]
Sex	MSERD	Conventional	-0.029	0.018	-1.640	0.101	[-0.063, 0.006]
	MSERD	Robust	-	-	-1.614	0.106	[-0.072, 0.007]
Medical payment method	MSERD	Conventional	-0.255	0.070	-3.641	<0.001	[-0.392, -0.118]
	MSERD	Robust	-	-	-3.238	0.001	[-0.409, 0.101]
Occupation	MSERD	Conventional	0.006	0.097	0.060	0.952	[-0.185, 0.197]
	MSERD	Robust	-	-	-0.083	0.934	[-0.237, 0.218]
Diagnostic coding	MSERD	Conventional	0.082	0.108	0.756	0.450	[-0.131, 0.294]
	MSERD	Robust	-	-	0.379	0.705	[-0.209, 0.310]

NOTE: MSERD iσ βandwidth that minimizes the MSE of the point estimator, where the bandwidth is the same on both sides of the cutoff

#### Regression discontinuity analysis

As shown in Table 7, local polynomial regression was performed with January 2019 as the cutoff point, using total hospitalization costs, comprehensive fees, diagnostic fees, treatment fees, consumption fees, and medicine fees as outcome variables.

**Table 7:** The influence of day-time surgical management mode on the cost of cancer inpatient treatment

Result variable	Model	Bandwidth	Parameter	Coef.	SD	z	*p*	95%CI
All cost	Unadjust	MSESUM	Conventional	-993.75	307.97	-3.227	0.001	[-1597.35, -390.144]
			Robust	-	-	-1.728	0.084	[-1299.63, 81.873]
	Adjust	MSESUM	Conventional	-1009.1	302.26	-3.334	0.001	[-1601.47, -416.649]
			Robust	-	-	-1.806	0.071	[-1304.55, 53.2864]
Comprehensive	Unadjust	MSESUM	Conventional	-292.75	13.616	-21.501	0.000	[-319.441, -266.066]
			Robust	-	-	-17.108	0.000	[-310.233, -246.455]
	Adjust	MSESUM	Conventional	-293.85	13.474	-21.809	0.000	[-320.26, -267.444]
			Robust	-	-	-17.262	0.000	[-311.227, -247.758]
Diagnosis	Unadjust	MSESUM	Conventional	-547.5	21.588	-25.361	0.000	[-589.811, -505.188]
			Robust	-	-	-19.712	0.000	[-574.408, -470.513]
	Adjust	MSESUM	Conventional	-508.3	25.095	-20.255	0.000	[-557.483, -459.113]
			Robust	-	-	-15.932	0.000	[-546.933, -427.103]
Treatment	Unadjust	MSESUM	Conventional	168.76	84.099	2.007	0.045	[3.92769, 333.59]
			Robust	-	-	3.218	0.001	[114.641, 471.79]
	Adjust	MSESUM	Conventional	495.29	89.545	5.531	0.000	[319.789, 670.8]
			Robust	-	-	6.532	0.000	[455.191, 845.504]
Consumption	Unadjust	MSESUM	Conventional	-400.49	25.325	-15.814	0.000	[-450.126, -350.855]
			Robust	-	-	-12.610	0.000	[-449.509, -328.571]
	Adjust	MSESUM	Conventional	-386.38	25.969	-14.879	0.000	[-437.281, -335.486]
			Robust	-	-	-11.785	0.000	[-433.837, -310.109]
Medicine	Unadjust	MSESUM	Conventional	-1518.7	186.61	-8.138	0.000	[-1884.42, -1152.94]
			Robust	-	-	-6.050	0.000	[-1905.16, -972.858]
	Adjust	MSESUM	Conventional	-1569.5	173.28	-9.058	0.000	[-1909.17, -1229.93]
			Robust	-	-	-6.676	0.000	[-1923.15, -1050.22]

Unadjusted: Only grouping variables were included (whether the day surgery management model was accepted); Adjusted for covariates including sex, age, occupation, diagnostic coding

MSESUM: βandwidth that minimizes the MSE of the sum of the regression coefficients

**Fig. 2:**
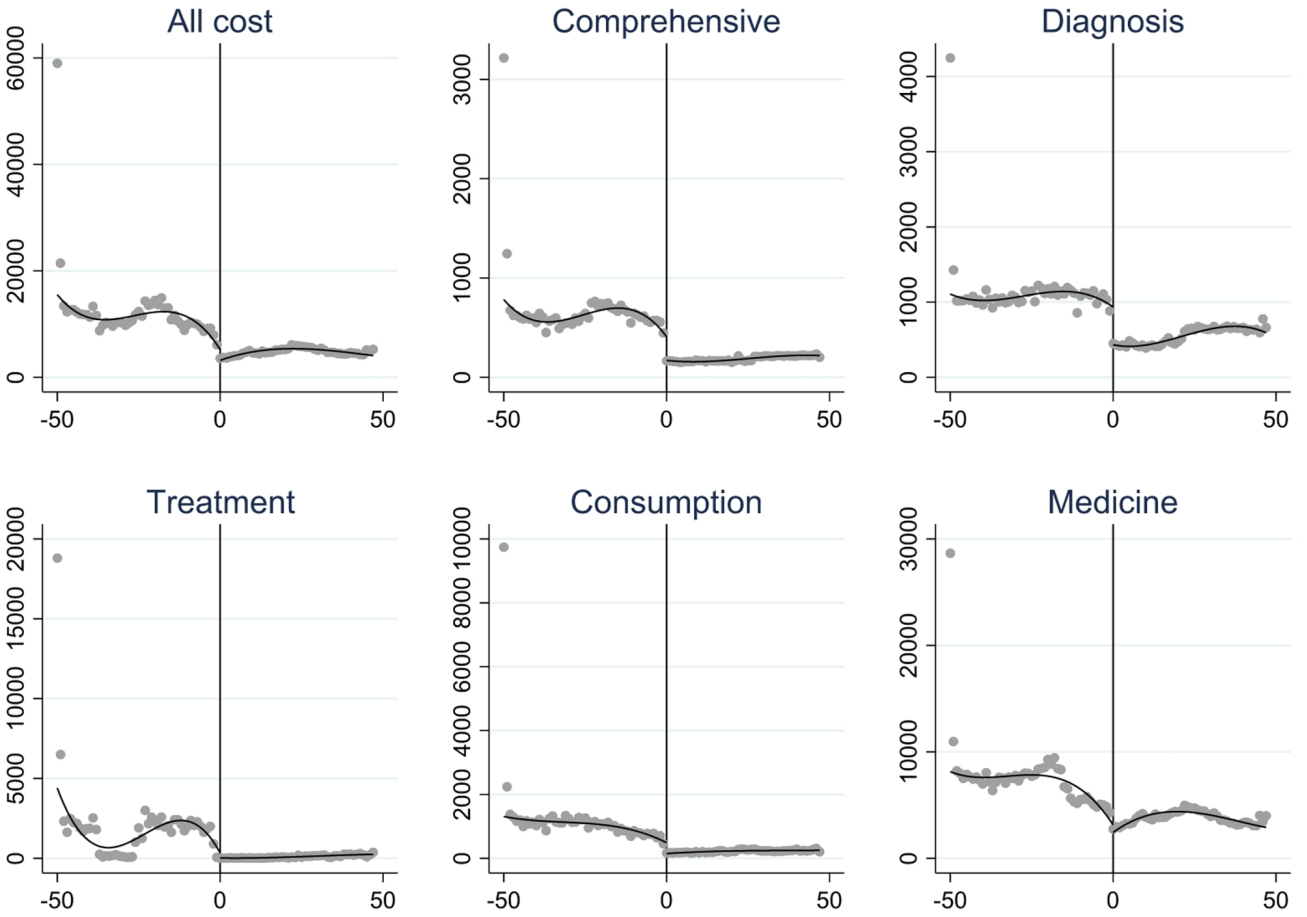
Cost discontinuity of cancer inpatient care. The vertical axis of each chart is the RMB cost, the horizontal axis is the month, 0 is January 2019, and a scale represents January

Covariates included in the polynomial regression model were all variables listed in Table 1, except for the medical payment method. The regression discontinuity analysis was conducted for models both with and without the inclusion of the control variables. Figure 2 displays regression discontinuity plots with fitted curves, revealing clear discontinuities at the January 2019 policy threshold across all six expense categories, indicating a significant post-policy reduction in each category. The results indicate that the implementation of the day-surgery did not have a statistically significant impact on total hospitalization costs for cancer treatment (*p* = 0.084), even after adjusting for covariates. In contrast, the analysis of the individual cost components revealed significant effects. Figure 2 illustrates the trends in each outcome variable over time, adjusted for covariates, demonstrating the discontinuities at the cutoff point and the model’s effect on specific cost components. The day-surgery management model significantly reduced comprehensive fees, diagnostic fees, consumption fees, and medicine fees, both before and after covariate adjustment. But treatment fees exhibited a significant increase under the day-surgery management model, regardless of whether covariates were included in the analysis.

### Robustness check

#### Sensitivity analysis for bandwidth selection

Using local polynomial regression analyses and alternative MSERD and MSETWO bandwidth selection methods, Table 8 confirms the significant effects of the day-surgery management model on comprehensive fees, diagnostic fees, consumable fees, and medication fees, indicating that our results are robust and not sensitive to the choice of bandwidth selection method.

**Table 8:** Effects of different bandwidth choices on the result variables

Result variable	Model	Parameter	Coef.	SD	z	*p*	95%CI
Comprehensive	MSERD	Conventional	-294.26	13.453	-21.874	0.000	[-320.631, -267.897]
		Robust	-	-	-18.144	0.000	[-314.741, -253.372]
	MSETWO	Conventional	-288.05	13.885	-20.745	0.000	[-315.266, -260.837]
		Robust	-	-	-16.897	0.000	[-307.768, -243.79]
Diagnosis	MSERD	Conventional	-496.02	27.636	-17.948	0.000	[-550.184, -441.852]
		Robust	-	-	-15.218	0.000	[-543.138, -419.196]
	MSETWO	Conventional	-510.98	25.241	-20.244	0.000	[-560.448, -461.505]
		Robust	-	-	-16.669	0.000	[-553.976, -437.408]
Treatment	MSERD	Conventional	606.43	95.664	6.339	0.000	[418.931, 793.926]
		Robust	-	-	7.253	0.000	[555.318, 966.548]
	MSETWO	Conventional	607.23	94.579	6.420	0.000	[421.855, 792.597]
		Robust	-	-	7.284	0.000	[553.06, 960.274]
Consumption	MSERD	Conventional	-331.28	29.002	-11.423	0.000	[-388.121, -274.436]
		Robust	-	-	-9.206	0.000	[-374.728, -243.17]
	MSETWO	Conventional	-374.93	26.54	-14.127	0.000	[-426.948, -322.913]
		Robust	-	-	-11.320	0.000	[-423.556, -298.535]
Medicine	MSERD	Conventional	-1311	207.3	-6.324	0.000	[-1717.29, -904.678]
		Robust	-	-	-4.625	0.000	[-1691.79, -684.647]
	MSETWO	Conventional	-1304.3	201.49	-6.473	0.000	[-1699.17, -909.341]
		Robust	-	-	-4.654	0.000	[-1675.68, -682.55]

Note: MSERD: Bandwidth that minimizes the MSE of the point estimator, where the bandwidth is the same on both sides of the cutoff; MSETWO: Bandwidth that minimizes the MSE of the point estimator, where the bandwidths are different on each side of the cutoff

#### Placebo test for the regression discontinuity point

To verify the robustness of the impact of the day surgery management model on hospitalization expenses, we conducted placebo tests using alternative cut-off points such as July 2018 and July 2019. The results are shown in Table 9. After selecting the MSE-optimal bandwidth and controlling for covariates, for the July 2018 cut-off point the comprehensive fees exhibited a significant positive shift (LATE = 46.245, *p* = 0.017), but the magnitude (+ 46.2 RMB) was much smaller than the original cut-off point LATE (-293.85 RMB). Other expense categories did not show statistical significance (*p* > 0.05). For the July 2019 cut-off point, diagnostic fees showed a significant negative effect (LATE = -98.628, *p* = 0.029), but the magnitude (-98.6 RMB) was much smaller than the original cut-off point LATE (-508.3 RMB). Medicine fees exhibited an abnormally large increase (LATE = 986.99, *p* < 0.001), which was directionally contradictory to the significant decrease observed at the original cut-off point. Other expense categories did not show statistical significance (*p* > 0.05). Further robustness tests were conducted on medicine fees using non-policy nodes such as January 2018 and January 2020. The treatment effects on medical expenses were found to be insignificant (January 2018 LATE = 266.01, *p* = 0.402; January 2020 LATE = -51.987, *p* = 0.708).

**Table 9:** Placebo test: Effects of different breakpoints

Result variable	New break	Parameter	Coef.	SD	z	*p*	95%CI
Comprehensive	07/2018	Conventional	46.245	19.297	2.397	0.017	[8.423, 84.066]
		Robust	-	-	3.648	0.000	[36.395, 120.935]
	07/2019	Conventional	5.117	4.466	1.146	0.252	[-3.637, 13.871]
		Robust	-	-	-1.218	0.223	[-14.887, 3.473]
Diagnosis	07/2018	Conventional	54.951	42.427	1.295	0.195	[-28.204, 138.105]
		Robust	-	-	1.153	0.249	[-38.792. 149.695]
	07/2019	Conventional	-98.628	45.141	-2.185	0.029	[-187.101, -10.154]
		Robust	-	-	-3.210	0.001	[-243.006, -58.745]
Treatment	07/2018	Conventional	1.292	241.86	0.0053	0.996	[-472.753, 475.338]
		Robust	-	-	0.0567	0.955	[-516.485, 547.25]
	07/2019	Conventional	9.767	12.539	0.779	0.436	[-14.809, 34.342]
		Robust	-	-	-6.667	0.000	[-152.122, -82.998]
Consumption	07/2018	Conventional	109.68	51.326	2.137	0.033	[9.082, 210.276]
		Robust	-	-	1.505	0.132	[-28.012, 213.436]
	07/2019	Conventional	2.4552	10.531	0.233	0.816	[-18.186, 23.096]
		Robust	-	-	-0.731	0.465	[-32.308, 14.753]
Medicine	07/2018	Conventional	-549.1	327.62	-1.676	0.094	[-1191.22, 93.018]
		Robust	-	-	-1.498	0.134	[-1339.22, 178.722]
	07/2019	Conventional	986.99	122.74	8.041	0.000	[746.425, 1227.55]
		Robust	-	-	7.667	0.000	[806.071, 1359.72]
	01/2018	Conventional	266.01	317.13	0.839	0.402	[-355.553, 887.574]
		Robust	-	-	0.826	0.409	[-452.937, 1112.22]
	01/2020	Conventional	-51.987	139.03	-0.374	0.708	[-324.49, 220.515]
		Robust	-	-	0.297	0.767	[-267.528, 362.909]

This series of tests indicates that the decreases in comprehensive fees (-RMB293.85) and diagnostic fees (-RMB508.3) observed at the original cut-off point (January 2019) were time-specific; medicine fees exhibited abnormal fluctuations in July 2019; and other major cost categories did not show systematic shifts at non-policy nodes. These tests support the robustness of the original results.

#### Sensitivity test for sample selection

**Fig. 3:**
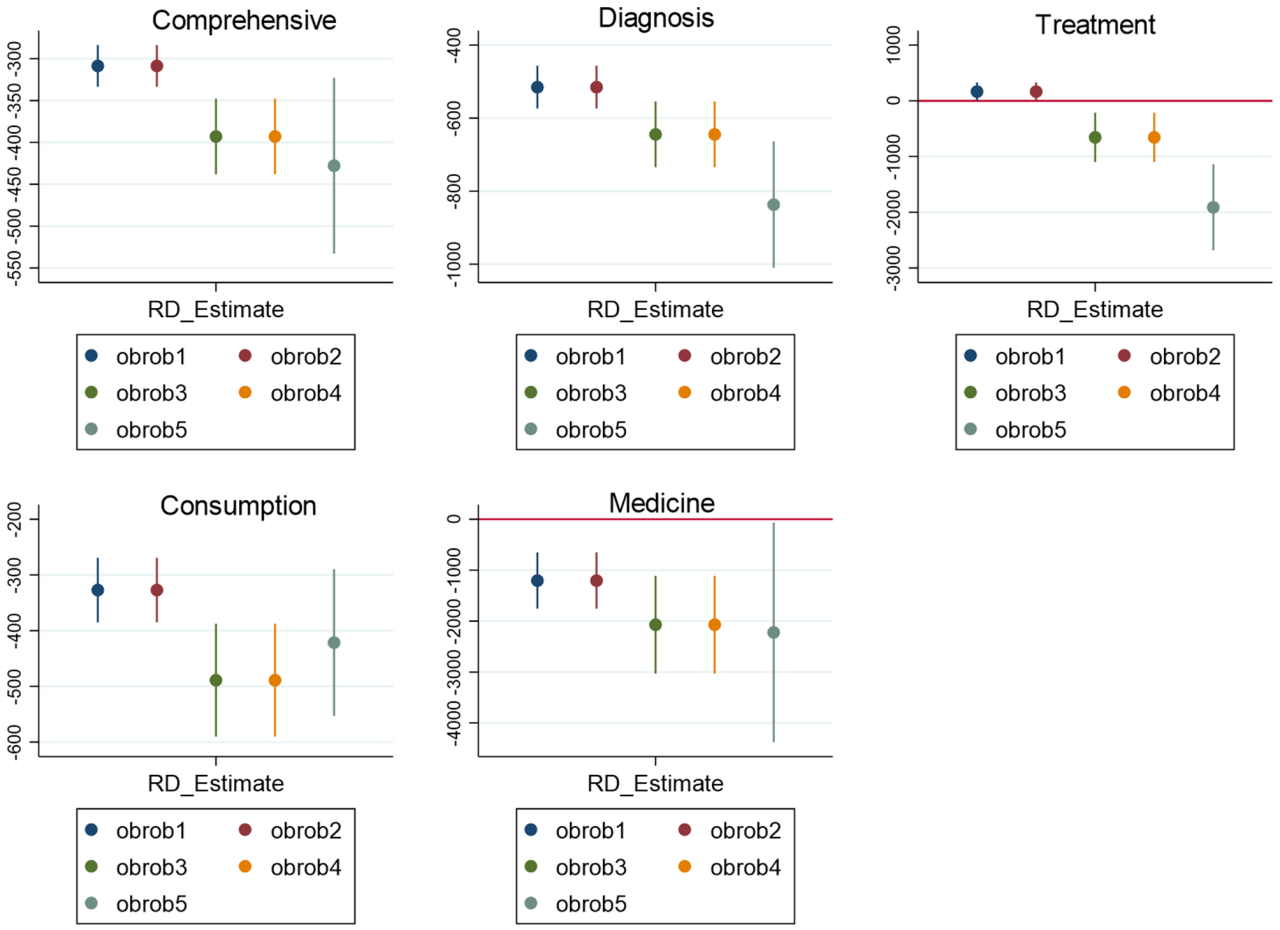
Donut effect. Obrob 1–5 corresponds to 1%-5% sample size around the deletion breakpoint. The fact that the vertical line does not cross the zero scale indicates that the result remains significant

To further assess the robustness of our results, a sensitivity test was conducted using the Donut Hole approach. This method addresses potential bias or manipulation near the discontinuity point, where samples very close to the threshold may be more susceptible to manipulation due to stronger incentives. The analysis evaluated the significance of the regression results by progressively removing samples closest to the discontinuity point. Specifically, 1%, 2%, 3%, 4%, and 5% of the samples nearest to the cutoff point were excluded from the analysis. Covariates were included in the model, and the MSESUM method was applied to select the optimal bandwidth. The significance of the five outcome variables was then re-evaluated. Figure 3 shows that even after the sequential removal of 1%-5% of the samples near the discontinuity point, the results for all five outcome variables remained significant. This suggests that there was no evidence of sample manipulation near the cutoff point.

#### Sensitivity analysis for length of stay adjustment

To elucidate the mechanism underlying the cost changes and address the potential mediating role of hospitalization duration, we conducted a sensitivity analysis by introducing length of stay (LOS) as a covariate in the RDD model. This analysis aims to distinguish whether the cost changes were driven by the reduction in hospital days or by changes in the daily intensity of medical services.

The results in Table 10 compare the baseline estimates with those adjusted for LOS. Controlling for LOS led to a structural shift in total hospitalization costs, changing from a statistically insignificant decrease (-RMB1009.1) in the baseline model to a significant increase (+ RMB564.7). Similarly, the treatment fees surged dramatically from +RMB495.3 to +RMB1614.2 when holding hospitalization time constant. Conversely, the cost-saving effects observed in comprehensive, diagnostic, consumables, and medicine fees were significantly attenuated when LOS was controlled. Notably, the reduction in medicine fees shrank from -RMB1569.5 to -RMB504.0, indicating that approximately two-thirds of the drug cost savings were dependent on shorter hospital stays.

**Table 10:** Impact of controlling for LOS across all cost categories

Outcome Variable	Model Specification	RD Estimate (Coeff.)	*P*-value
Total Costs	Baseline	-1009.1	0.071
	Controlled for LOS	564.7***	0.002
Comprehensive Fees	Baseline	-293.9***	0.000
	Controlled for LOS	-126.4***	0.000
Diagnostic Fees	Baseline	-508.3***	0.000
	Controlled for LOS	-220.1***	0.000
Treatment Fees	Baseline	495.3***	0.000
	Controlled for LOS	1614.2***	0.000
Consumable Fees	Baseline	-386.4***	0.000
	Controlled for LOS	-92.8***	0.000
Medicine Fees	Baseline	-1569.5***	0.000
	Controlled for LOS	-504.0***	0.003

Note: ‘Baseline’ refers to the original model specification; ‘Controlled for LOS’ includes Length of Stay as a covariate. Significance levels: * *p* < 0.1, ** *p* < 0.05, *** *p* < 0.01

These findings reveal that the day-surgery model entails higher daily resource intensity, as evidenced by the spike in treatment costs when time is held constant. The reduction in hospitalization duration (LOS) was confirmed as the primary mechanism driving the savings in comprehensive and material costs, masking the increased technical intensity of the day-surgery procedures.

## Discussion

Using a time-based regression discontinuity design, we addressed the gap in the literature on the impact of the day-surgery management model on cancer hospitalization costs in China. Since national day-surgery policy is implemented at the province and city level, we undertook a case study in Ningxia.

While the regression discontinuity analysis found no statistically significant effect of the day-surgery management model on total cancer hospitalization costs (*p* = 0.084), specific cost components revealed significant reductions, including comprehensive fees, diagnostic fees, consumption fees, and medicine fees. Our sensitivity analysis in Table 10 provides critical insights into the mechanism of these changes. When controlling for LOS, the reduction in comprehensive fees fell by over 50% (from -RMB293.9 to -RMB126.4), and the total hospitalization costs shifted from a statistically insignificant decrease to a significant increase. This confirms that the cost-saving effect of the day-surgery model is predominantly mediated by the reduction in LOS. Shortening hospital stays translate into reduced bed occupancy, lowering all the costs associated with hospitalization [22]. Also, day surgery leads hospitals to focus on quick assessments and management before and after surgery, reducing unnecessary tests and treatments, which lowers diagnostic fees and consumption fees [23].

Treatment fees associated with the day-surgery model increased significantly, highlighting the importance of cost containment measures. This shift of costs is a pattern observed internationally, where the primary savings from reduced length of stay can be partially or fully offset by the intensity of services delivered on the day of surgery. The economic outcome is critically dependent on the local payment model. Hospitals should formulate strategies to optimize surgical efficiency without adding an additional financial burden to patients. Efficient and successful implementation of the day-surgery model requires adequate medical resources, including operating rooms, equipment and trained staff. Hospitals should strategically allocate resources to support day-surgery while maintaining high quality care.

The day-surgery treatment costs rise was associated with the higher demand for advanced technologies and equipment in day surgery, as well as enhanced post-operative follow-up and care requirements. Our study highlights the need for hospitals to strike a balance between surgical efficiency and the overall hospitalization costs for patients. Studies confirm a universal policy aim to strengthen day-surgery to increase health system efficiency, often employing specific financial incentives within broader payment reforms. Specifically, the reimbursement for each oncological day-case should be prospectively discounted by the province-specific percentage reduction in length-of-stay relative to the inpatient counterpart, and the resulting amount split into a fixed 85% core payment and a conditional 15% reserve. This reserve would be disbursed subsequently based on performance metrics, such as meeting clinical outcome benchmarks, achieving patient satisfaction targets, and demonstrating cost-effectiveness. Implementation could involve integrating this tiered payment structure into existing Diagnosis-Intervention Packet (DIP) or Diagnosis-Related Group (DRG) frameworks. Developing reasonable cost control strategies is essential if the day surgery model is to gain traction.

The p-value of medical payment methods at the breakpoint was discontinuous, but after conducting a fuzzy breakpoint regression analysis, the results indicated continuity (*p* = 0.417). This difference may arise from the varying adaptability of overall costs under different medical payment models. For example, the traditional fee-for-service model may lead physicians to prefer higher-cost treatment options, while the payment methods under the day surgery management model might incentivize doctors to focus on cost-reduction strategies [24]. Also, different medical payment methods may influence the cost-effectiveness of the day surgery management model. In some countries or regions, fixed payments or capitation payment models encourage hospitals to adopt more efficient management strategies to reduce overall healthcare costs [25]. To provide more targeted recommendations for policymakers, we recommend future research explores the operation of different medical payment schemes for day-surgery.

Our study has several limitations. First, the case study was conducted in Yinchuan, Ningxia, which may limit the generalizability of the findings to other regions in China. Variations in provincial and local healthcare infrastructure, patient demographics, and implementation of national day-surgery policies could significantly influence the applicability of our results to other cities and provinces. Second, the reliance on hospital information system data may restrict the depth of insights into patient experiences and outcome measures. Additionally, patient acceptance and perceptions regarding day surgery were not assessed, despite evidence suggesting that a substantial proportion of Chinese patients express concerns about postoperative complications (up to 75%) and inadequate recovery knowledge (53%)^8^. Furthermore, the observational period coincides with the COVID-19 pandemic, a significant external shock that may have concurrently influenced hospitalization patterns, resource allocation, and cost structures. Although our RDD design aims to isolate the policy effect at a specific cutoff, the pandemic’s broader systemic disruptions could represent confounding factors that are challenging to fully disentangle from the policy impact. Finally, while the regression discontinuity design was employed, alternative methodologies, such as randomized controlled trials (RCTs), qualitative studies, or econometric analyses, could provide stronger evidence and deeper insights into the causal relationships and long-term cost-effectiveness of day surgery. Future research should employ longitudinal designs to track post-discharge costs and patient quality of life, ensuring that the cost savings from shorter stays do not come at the expense of patient safety.

## Conclusion

Using a regression discontinuity design, our study evaluated the impact of the day-surgery management model on cancer treatment costs in Ningxia, China. The findings indicate that while the day-surgery model did not significantly reduce total hospitalization costs, it led to notable decreases in comprehensive, diagnostic, consumption, and medicine fees. Our sensitivity analysis robustly confirmed that these savings were primarily mediated by shorter hospital stays. However, once the length of stay was controlled, the significant increase in treatment fees revealed the high daily resource intensity of the day-surgery model.

These results underscore the complexity of day-surgery economics: efficiency gains from faster turnover were partially offset by technology-driven cost escalations. Simply “improving efficiency” is insufficient. To enhance the financial sustainability of day-surgery reforms, policymakers and hospitals should implement targeted cost-containment strategies, such as adopting bundled payment methods calibrated for day surgery and enforcing standardized clinical pathways to curb excessive treatment intensity. Future research should explore city, province and regional variations; adopt alternative methodologies, such as randomized controlled trials or longitudinal studies; and implement mixed-methods approaches, combining quantitative data with qualitative patient and provider perspectives. Comparative studies across diverse healthcare systems would also help identify best practices and scalable solutions for optimizing day-surgery outcomes globally.

## Data Availability

The data presented in this study are available on request from the corresponding author.
